# SiO_2_/ZnO Composite Hollow Sub-Micron Fibers: Fabrication from Facile Single Capillary Electrospinning and Their Photoluminescence Properties

**DOI:** 10.3390/nano7030053

**Published:** 2017-02-24

**Authors:** Guanying Song, Zhenjiang Li, Kaihua Li, Lina Zhang, Alan Meng

**Affiliations:** 1 Key Laboratory of Polymer Material Advanced Manufacturings Technology of Shandong Provincial, College of Electromechanical Engineering, College of Sino-German Science and Technology, Qingdao University of Science and Technology, Qingdao 266061, Shandong, China; songgy80@126.com (G.S.); likh_199109@163.com (K.L.); zhln_qust@163.com (L.Z.); 2 State Key Laboratory Base of Eco-chemical Engineering, College of Chemistry and Molecular Engineering, Qingdao University of Science and Technology, Qingdao 266042, Shandong, China; alanmengqust@163.com

**Keywords:** SiO_2_/ZnO, hollow sub-micron fibers, electrospinning, mechanism, photoluminescence

## Abstract

In this work, SiO_2_/ZnO composite hollow sub-micron fibers were fabricated by a facile single capillary electrospinning technique followed by calcination, using tetraethyl orthosilicate (TEOS), polyvinylpyrrolidone (PVP) and ZnO nanoparticles as raw materials. The characterization results of the scanning electron microscopy (SEM), transmission electron microscopy (TEM), X-ray diffraction (XRD) and Fourier transform infrared spectroscopy (FT-IR) spectra indicated that the as-prepared composite hollow fibers consisted of amorphous SiO_2_ and hexagonal wurtzite ZnO. The products revealed uniform tubular structure with outer diameters of 400–500 nm and wall thickness of 50–60 nm. The gases generated and the directional escaped mechanism was proposed to illustrate the formation of SiO_2_/ZnO composite hollow sub-micron fibers. Furthermore, a broad blue emission band was observed in the photoluminescence (PL) of SiO_2_/ZnO composite hollow sub-micron fibers, exhibiting great potential applications as blue light-emitting candidate materials.

## 1. Introduction

One-dimensional hollow nanostructure, as a specific one-dimension morphology, has attracted considerable attentions due to its wide application prospect in many fields such as electrochemical energy storage, biomedical drugs delivery, sensors, chemical reactions in nanoscale [1,2,3,4,5,6]. Up to now, various methods have been developed to fabricate one-dimensional hollow nanostructures, e.g., self-assembly technique [7], template-directed synthesis [8,9]. Electrospinning has been actively exploited as a simple and versatile method for fabricating various one-dimensional nanostructures, including nanofibers [10,11,12], core-shell composite [13], porous structure [14] etc. Especially, the electrospinning method is considered powerful tool for preparing one-dimensional hollow nanostructure [15]. Recent research works have shown that 1D tubular nanostructure is facilely fabricated by using the two-capillary coaxial electrospinning technique. When this technique is used, two kinds of different solutions are ejected simultaneously through a coaxial spinneret with two-capillaries, then the core of the fibers is selectively removed, and hollow nanofibers are formed. It has been reported that various types of materials hollow nanofibers including TiO_2_ [16,17], SiO_2_ [18], PVDF/PVA [19], PMMA [20] have been fabricated by this coaxial electrospinning technique. It is worth noting that there are still some problems in obtaining high-quality hollow nanofibers by coaxial electrospinning, such as the selection of the inner solvent and the control of electrospinning parameters, and so on. On the other hand, the electrospinning technique with a single capillary has been reported to be developed for fabricating hollow nanofibers by few researchers. Choi’s group and Shao’s group reported that the SnO_2_ hollow nanofibers [21], polymer hollow nanofibers [22] and ZnO hollow nanofibers [23] were prepared by electrospinning with a single capillary. However, the fabrication of the hybrid materials hollow nanofibers by using this single capillary electrospinning technique has been rarely investigated until now.

SiO_2_ hollow nanofibers are of growing interest because of their unique properties. Zhan et al. [18] reported that SiO_2_ hollow fibers with hierarchical walls were fabricated by the sol-gel combined two-capillary spinneret co-electrospinning technique, using triblock copolymer as the porous directing agent. Zygmunt et al. [24] used the vanadium oxide hydrate as template to synthesize the SiO_2_ nanotubes with good chemical purity, a well defined morphology and high aspect ratio. An et al. [25] reported the synthesis of hollow SiO_2_ nanowires via the electrospinning technique. Ogihara et al. [26] synthesized the SiO_2_ nanotubes by the hydrolysis of tetraethyl orthosilicate (TEOS) with carbon nanofibers as templates. In addition, the application of SiO_2_ nanotubes as nanoscale reactors was also investigated. Generally, it is believed that SiO_2_ composite hollow nanofibers might possess the improved properties and the extended applications. However, SiO_2_-basedcomposite hollow nanofibers have not been reported to the best of our knowledge.

In the present work, a simple single-spinner electrospinning technique was developed to fabricate the composite hollow sub-micron fibers consisting of amorphous SiO_2_ and hexagonal wurtzite structure ZnO, using tetraethyl orthosilicate (TEOS), polyvinylpyrrolidone (PVP) and ZnO nanoparticles as raw materials. The as-prepared products revealed uniform tubular structure with the diameters of 400–500 nm and the wall thickness of 50–60 nm. The gases generated and the directional escaped growth mechanism was put forward. The obtained SiO_2_/ZnO composite hollow sub-micron fibers display unique blue emission properties. Compared with the traditional method for synthesizing the composite tubular nanostructure, the single capillary electrospinning technique is remarkably simple and efficient. This research work not only provides a new route for synthesizing SiO_2_/ZnO composite hollow sub-micron fibers but also offers a general reference for other materials composite hollow nanostructures synthesis.

## 2. Experimental Section

### 2.1. Materials

Polyvinylpyrrolidone (PVP, M_w_ = 1,300,000) was purchased from Tianjin Bodi Chemical Co., Ltd., Tianjin, China. Tetraethyl orthosilicate (TEOS) was purchased from Tianjin Basifu Chemical Co., Ltd., Tianjin, China. Acetic acid (36 wt. %) was purchased from Sinopharm Chemical Reagent Co., Ltd., Beijing, China. The above chemical reagents were analytical grade, without further purification. The ZnO nanoparticles with the diameter of about 50 nm were prepared by our previously reported synthesis method [27].

### 2.2. Preparation of SiO_2_/ZnO Composite Hollow Sub-Micron Fibers

In a typical procedure for preparing SiO_2_/ZnO composite hollow sub-micron fibers, firstly, 1 mL TEOS was dissolved in 3 mL ethanol, and 0.25 g PVP and 0.5 mL acetic acid were dissolved in the TEOS ethanol solution at room temperature with magnetic stirring for 60 min. Secondly, 0.1 g ZnO nanoparticles were added into the above solution and ultrasonically dispersed for 2 h to form the homogeneous precursor solution. Thirdly, the as-prepared precursor solution was poured into a glass syringe with a capacity of 5 mL equipped with a 23 gauge needle. The relevant parameters of electrospinning were as follows: the feeding rate of the solution was adjusted at 1 mL·h^−1^ by the syringe pump. The applied voltage and the distance between the needle and collector were 25 kV and 15 cm, respectively. The SiO_2_/ZnO composite hollow sub-micron fibers were deposited on an aluminum foil collector during the electrospinning procedure. Finally, to decompose the PVP completely, the as-spun sub-micron fibers were calcined at 550 °C for 2 h with the heating rate of 10 °C/min. The SiO_2_/ZnO composite hollow sub-micron fibers were achieved. In addition, as a contrast sample, SiO_2_ sub-micron fibers were prepared by the same method described previously. The only difference is the absence of ZnO nanoparticles in raw material.

### 2.3. Characterization

The morphologies of the as-synthesized products were characterized by a field emission scanning electron microscopy equipped with an energy-dispersive X-ray spectrometer (EDS) unit (FESEM; S4800, Hitachi, Tokyo, Japan) and a transmission electron microscope (TEM; JEM-2000EX, JEOL, Tokyo, Japan). X-ray diffraction (XRD) pattern was recorded by using a D/max-2400 X-ray diffractometer (Rigaku, Tokyo, Japan) at room temperature. Infrared absorbance spectrum (IR) measurement was performed in a Nicolet FT-IR spectrophotometer (FT-IR; Nicolet-360, Madison, WI, USA). In addition, the photoluminescence spectral measurements were performed in a Hitachi F-4500 fluorescence spectrophotometer using a xenon lamp at room temperature (F-4500, Hitachi, Tokyo, Japan).

## 3. Results and Discussion

**Figure 1:**
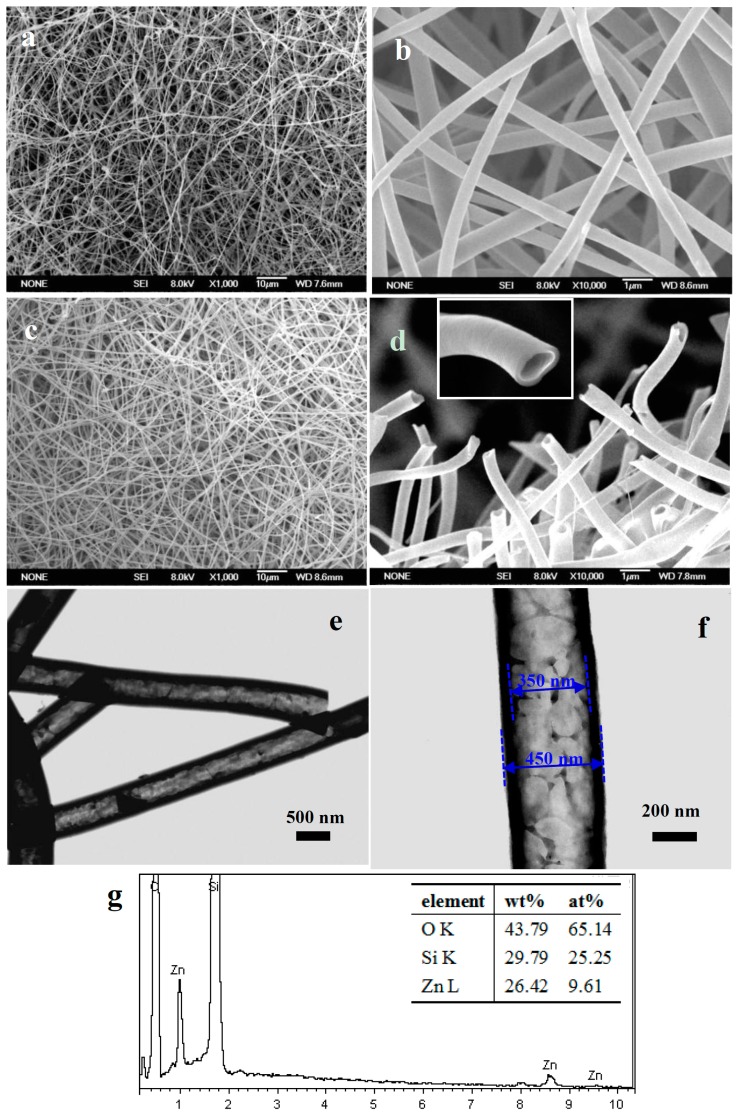
(**a**) low magnification and (**b**) high magnification scanning electron microscopy (SEM) images of as-spun precursor sub-micron fibers; (**c**) low magnification and (**d**) high magnification SEM images of SiO_2_/ZnO composite hollow sub-micron fibers and the cross-section of a single hollow nanofiber (the inset in (**d**)); (**e**) low magnification and (**f**) high magnification TEM images of SiO_2_/ZnO composite hollow sub-micron fibers; (**g**) Energy-dispersive X-ray spectroscopy of the SiO_2_/ZnO composite hollow sub-micron fibers.

Figure 1a,b showed the SEM images of the as-prepared precursor sub-micron fibers. It could be observed that large-scale sub-micron fibers with dozens of micrometers in length have been achieved. These randomly oriented sub-micron fibers had smooth surface and uniform diameter in the range of 500–600 nm. Figure 1c,d showed SEM images of the sub-micron fibers obtained by calcining precursor nanofibers at 550 °C. The obtained sub-micron fibers revealed uniform diameters and dozens of micrometers in length. The cross sections in Figure 1d clearly showed the sub-micron fibers are hollow structure. The outer diameters of SiO_2_/ZnO composite hollow sub-micron fibers were 400–500 nm, which were slight smaller than the diameter of precursor nanofiber. The reduction of diameters resulted from the decomposition of PVP during the calcinations process. High-magnification SEM image of a single SiO_2_/ZnO hollow sub-micron fibers (inset in Figure 1d) showed that the surface was slightly rough. Figure 1e,f showed the typical TEM images of SiO_2_/ZnO composite hollow sub-micron fibers at low and high magnifications, respectively. These TEM images further confirmed that the as-prepared products possess 1D hollow structure with the uniform large inner diameters of about 300–400 nm. The wall thickness was about 50–60 nm. The ZnO nanoparticles with diameters of 50–80 nm could be clearly seen in the wall of the composite hollow sub-micron fibers. In addition, some larger accumulations consisted of the smaller ZnO nanoparticles.

Figure 1g was the typical energy dispersive X-ray spectra (EDS) recorded from the SiO_2_/ZnO composite hollow sub-micron fibers, which showed the presence of the Si, O and Zn elements. We can deduce that the wall of the composite hollow sub-micron fibers consist of SiO_2_ and ZnO.

**Figure 2:**
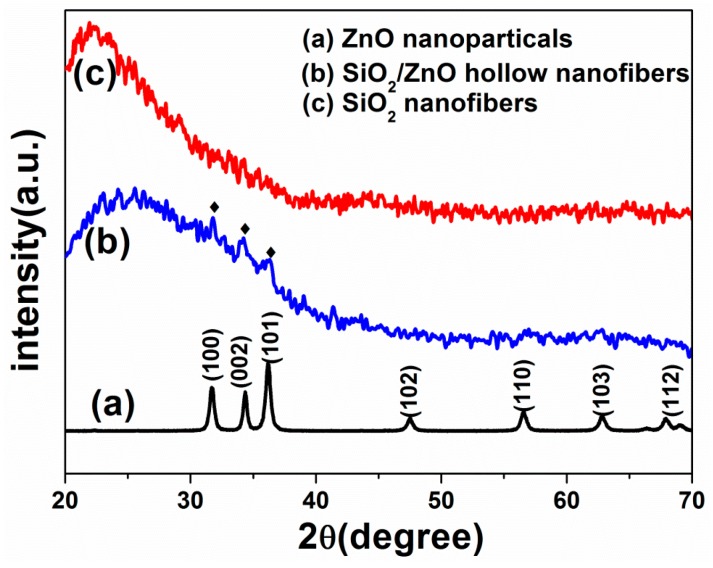
X-ray diffraction (XRD) patterns of (**a**) ZnO nanoparticles, (**b**) SiO_2_/ZnO composite hollow sub-micron fibers and (**c**) pure SiO_2_ sub-micron fibers.

XRD patterns of self-prepared ZnO nanoparticles, as-prepared SiO_2_ sub-micron fibers and SiO_2_/ZnO composite hollow sub-micron fibers were shown in Figure 2. The curve (a) with typical characteristic peaks at 31.9°, 34.6°, 36.5°, 47.7°, 56.8°, 63.1°, 68.1° can be indexed to (100), (002), (101), (102), (110), (103), (112) planes of the hexagonal wurtzite ZnO, which were consistent with the values in the standard card (JCPDS 36-1451). The curve (c) was XRD patterns of as-prepared SiO_2_ sub-micron fibers, showing an amorphous broad peak around 22°. The curve *b* was XRD pattern of as-prepared SiO_2_/ZnO composite hollow sub-micron fibers. The broad peak around 22° originated from the amorphous SiO_2_. Three diffraction peaks at 31.9°, 34.6°, 36.5° can be identified to (100), (002), (101) planes of the hexagonal wurtzite ZnO, which were in good agreement with the standard card (JCPDS 36-1451). Thus, the results of XRD pattern proved that SiO_2_/ZnO composite hollow sub-micron fibers consisted of the amorphous SiO_2_ and crystallized ZnO.

**Figure 3:**
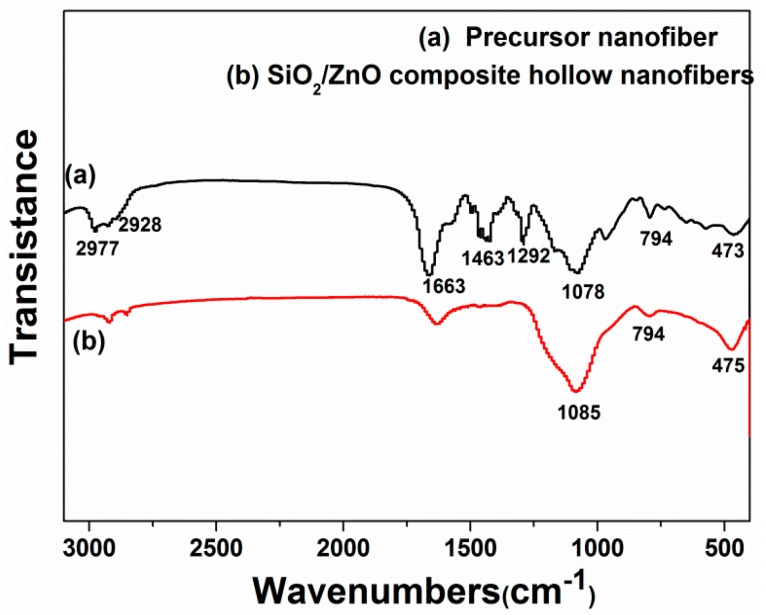
Fourier transform infrared spectroscopy (FT-IR) spectra of (**a**) the precursor sub-micron fibers and (**b**) SiO_2_/ZnO composite hollow sub-micron fiberscalcined at 550 °C.

Figure 3 displayed the FT-IR spectra of as-spun precursor sub-micron fibers and SiO_2_/ZnO composite hollow sub-micron fibers. For the spectrum of the precursor sub-micron fibers (curve (a) in Figure 3), strong peaks at around 1663, 1463 and 1292 cm^−1^ could obviously be observed, which were attributed to C=O, C=C and C–N stretching vibration of PVP [23]. The peaks at around 2977 and 2928 cm^−1^ should be assigned to the aliphatic CH group vibrations of CH_2_ absorption of PVP [23]. However, for the spectrum of SiO_2_/ZnO composite hollow sub-micron fibers (curve (b) in Figure 3), it was found that the characteristic peaks of PVP had disappeared, which indicated that the PVP could be removed completely from the precursor sub-micron fibers after calcination. The obvious peaks at around 1085, 794 cm^−1^ in the spectrum (b) were attributed to the stretching vibration of amorphous SiO_2_ [28]. The peak at around 475 cm^−1^ attributed to vibration of Zn–O bonds, indeed confirming the existence of ZnO [23]. Moreover, it can be found that the intensity of Zn–O bonds peak was stronger in SiO_2_/ZnO composite hollow sub-micron fibers than that of the as-spun precursor sub-micron fibers, revealing that the crystallinity of the ZnO nanoparticles have been improved after calcination at 550 °C.

**Figure 4:**
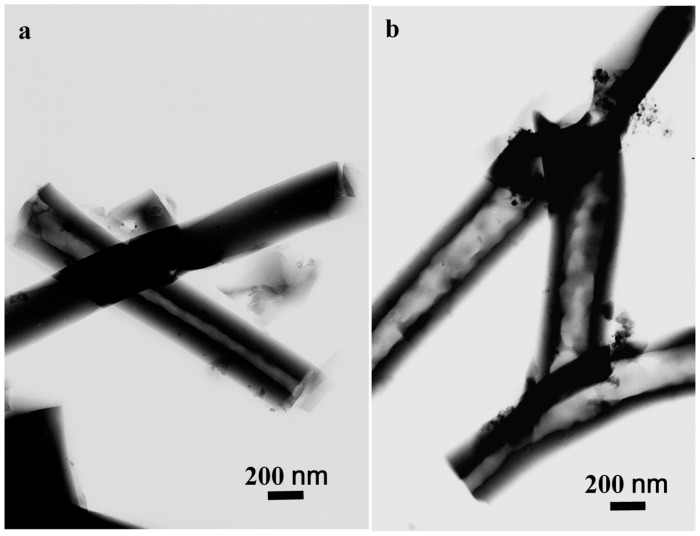
Transmission electron microscopy (TEM) images of (**a**) as-prepared SiO_2_ sub-micron fibers with both hollow and solid structures; (**b**) SiO_2_/ZnO composite hollow sub-micron fibers.

To elucidate the growth mechanism of the SiO_2_/ZnO composite hollow sub-micron fibers, the different dosages of ZnO in the raw material solution had been investigated, which is illustrated in the Supplementary Materials. The results shows that the optimum dosage of ZnO was 0.1 g in 1 mL TEOS. Figure 4 was the TEM images of the obtained SiO_2_ sub-micron fibers and SiO_2_/ZnO composite hollow sub-micron fibers synthesized under the optimized dosage of ZnO. As can be seen in Figure 4a, when the ZnO nanoparticles were absent from the raw material, both the hollow and the solid SiO_2_ sub-micron fibers were found simultaneously in as-prepared products. When the optimum dosage of ZnO was added to the raw materials solution, all the SiO_2_/ZnO composite hollow sub-micron fibers displayed hollow structure, as shown in Figure 4b. Moreover, the inner diameters of as-prepared SiO_2_/ZnO composite hollow sub-micron fibers were much larger than those of SiO_2_ hollow sub-micron fibers. All these results indicated that ZnO nanoparticles played an important role in forming SiO_2_/ZnO composite hollow sub-micron fibers.

On the basis of the above results, the possible gases generated and the directional escaped growth mechanism were proposed. During the calcination process, PVP became viscous and the fibers were plasticized with the increase of temperature, carbon monoxide and other gases were generated due to the PVP decomposition. Meanwhile, ethanol and water inside the fibers were also evaporated rapidly. All the gases escaped to the outside of the fibers through two pathways: along the axial or radial directions of fibers. The morphology of the formed fibers was determined by the ratio between the directional escaped rate and the generated rate of gases during the calcination process of precursor sub-micron fibers. If the radial direction escaped rate of gases was larger than the generated rate of gases inside the fibers, all the gases escaped along the radial direction of the fibers, no gases released along the axial direction of the fibers, thus the solid sub-micron fibers were formed. If the radial direction escaped rate of the gases was smaller than the generated rate of gases inside the fibers, there will be a part of gases escaped along the axial direction of fibers, resulting in the hollow sub-micron fibers. So that, in the case of SiO_2_ sub-micron fibers synthesis, the radial direction escaped rate and the generated rate of gases inside the fibers kept approximate equilibrium during the calcination process, large amount of the gases escaped along the radial pathway of fibers and small amount of the gases along the axial direction. As a result, the hollow nanofibers with relative smaller inner diameters and the solid sub-micron fibers appeared in as-prepared SiO_2_ sub-micron fibers.

In the case of the synthesizing SiO_2_/ZnO composite hollow sub-micron fibers, the ZnO nanoparticles could facilitate the denser layer forming at the fibers surface. During the calculation process, as the PVP gradually became viscous and decomposed, ZnO nanoparticles dispersed in the precursor fibers would move and gather drove by the surface energy decrease. At the same time, the amorphous SiO_2_ was generated from the pyrolysis of TEOS. It was worth mentioning that, the existing ZnO nanoparticles could serve as the direct growth position for amorphous SiO_2_, which would facilitated the amorphous SiO_2_ fast growth. The continuous growing amorphous SiO_2_ should fill quickly the interval among the ZnO nanoparticles or wrapped the ZnO nanoparticles. As a result, a dense layer consisted of amorphous SiO_2_ and ZnO nanoparticles was formed at the fiber surface, which would hinder the radial direction escaping of the gases inside the fibers. Therefore, large amount of gas escaped mainly along the axial direction pathway. The SiO_2_/ZnO composite hollow nanofibers with larger inner diameters were fabricated.

**Figure 5:**
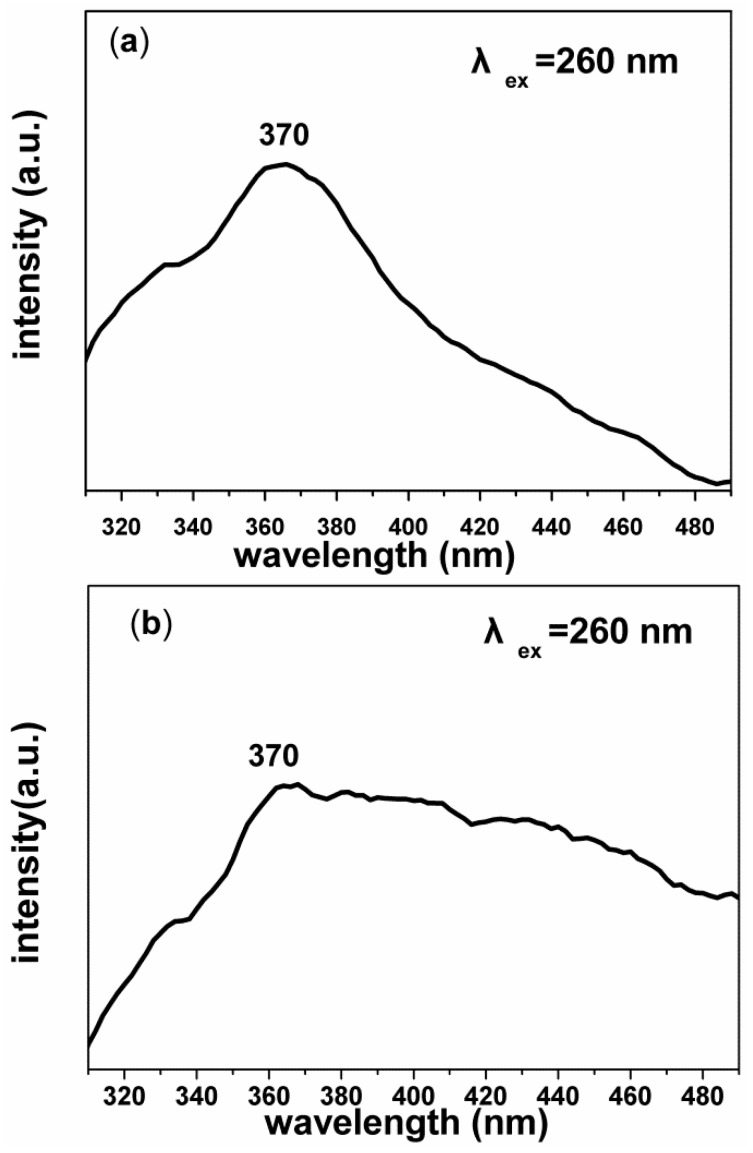
Photoluminescence spectrum of (**a**) SiO_2_ sub-micron fibers and (**b**) SiO_2_/ZnO composite hollow sub-micron fibers under excitation at 260 nm (λ_ex_ = 260 nm).

Figure 5 displayed the PL spectrum of the pure SiO_2_ sub-micron fibers and SiO_2_/ZnO composite hollow sub-micron fibersat room temperature under excitation at 260 nm. As was shown in Figure 5a, the pure SiO_2_ sub-micron fibers displayed a broad ultraviolet light emission band with a maximum around 370 nm, which may be attribute to the ≡Si−O−O−Si≡ and ≡Si−Si≡ defects emission of SiO_2_, which were in agreement with previous studies [29,30]. Compared to the SiO_2_ sub-micron fibers, besides the emission band at ultraviolet light, the emissionspectrum of SiO_2_/ZnO composite hollowsub-micron fibers (Figure 5b) presented an obviously enhanced broad blue emission band.

The enhancement of the blue emission intensity might originate from two factors. On the one hand, the radiative recombination of trapped electron from shallow donor levels created by various oxygen vacancies in the valence band of ZnO may lead to the blue emission [31,32,33]. On the other hand, it might be due to the interface defect of ZnO and SiO_2_ [33,34,35]. During the calcination process, with the increase of ZnO crystallization degree and the compounding of ZnO and SiO_2_, the local stress must be released and large numbers of defect states will present at the interface between the SiO_2_ and ZnO, which leads to an enhanced recombination emission.

## 4. Conclusions

In summary, the improved single capillary electrospinning technique was developed for successfully synthesizing SiO_2_/ZnO composite hollow sub-micron fibers. The obtained SiO_2_/ZnO composite hollow sub-micron fibers showed uniform hollow structure with the outer diameter of 400–500 nm and the wall thickness of 50–60 nm. The possible gases generated and the directional escaped growth mechanism was put forward to explain the formation of the composite hollow sub-micron fibers. The unique photoluminescence of SiO_2_/ZnO composite hollow sub-micron fibers exhibited great potential application in the field of the blue light-emitting photoelectric device. Moreover, the unique hollow sub-micron fibers structure may be applied in catalysts, gas storage, drug release, sensing, and environmental protection. Further studies will be carried out in the future.

## Supplementary Materials

The following are available online at http://www.mdpi.com/2079-4991/7/3/53/s1, Figure S1: Transmission electron microscopy (TEM) images of the products under different ZnO dosage in 1 mL TEOS. (**a**) 0 g; (**b**) 0.05 g; (**c**) 0.1 g; (**d**) scanning electron microscopy (SEM) image of the precursor fibers when the ZnO dosage was 0.15 g.
