# Supplementary material for: SiO2/ZnO Composite Hollow Sub-Micron Fibers: Fabrication from Facile Single Capillary Electrospinning and Their Photoluminescence Properties
Source: Nanomaterials (Basel). 2017 Feb 24;7(3):53. doi: 10.3390/nano7030053 (PMC5388155; doi:10.3390/nano7030053)
Supplement: Supplementary file 1 [file nanomaterials-07-00053-s001.pdf]

Supplementary Materials: SiO_2/ZnO Composite Hollow Sub-Micron Fibers: Fabrication from Facile Single Capillary Electrospinning and Their Photoluminescence Properties

Guanying Song, Zhenjiang Li, Kaihua Li, Lina Zhang and Alan Meng

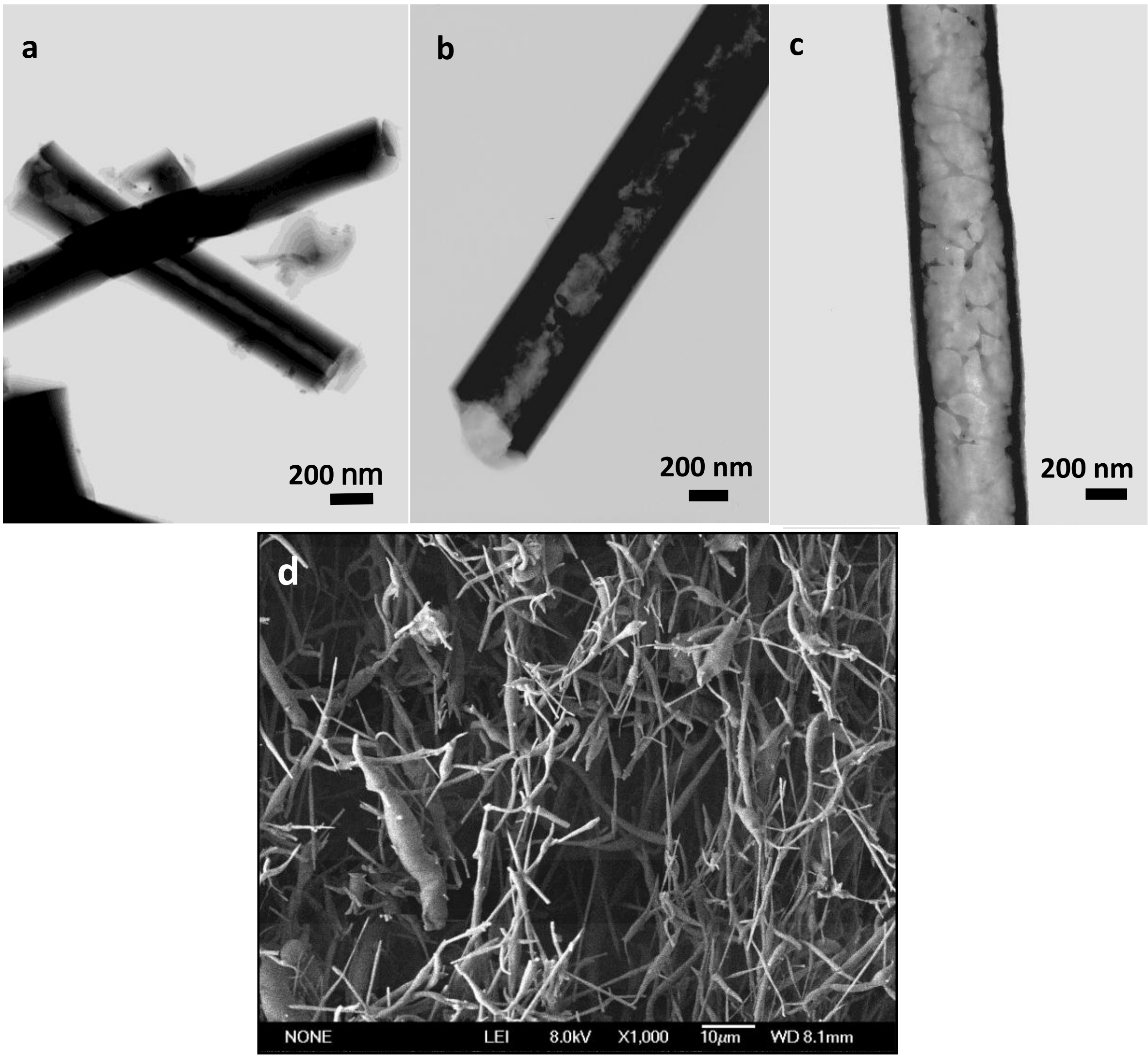

Figure S1. Transmission electron microscopy (TEM) images of the products under different ZnO dosage in 1 mL TEOS. (**a**) 0 g; (**b**) 0.05 g; (**c**) 0.1 g; (**d**) scanning electron microscopy (SEM) image of the precursor fibers when the ZnO dosage was 0.15 g.

The dosage of ZnO added in the raw material solution had an effect on the morphology of the products. The influence of the different ZnO dosage (0 g, 0.05 g, 0.1 g, 0.15 g) in 1 mL TEOS on the products morphology was investigated. And the corresponding TEM and SEM images are shown in Figure S1.

From Figure S1a,b, it can be seen that when there was no ZnO or lower ZnO dosage (0.05 g) in raw material solution, the products were predominant solid nanofibers and small amount of hollow nanofibers with relative smaller inner diameters. When the dosage of ZnO in raw material solution was 0.1g, all the products were uniform hollow fibers with larger inner diameters, as shown in Figure S1c. When the dosage of ZnO was excessive (0.15 g), the uniform and longer fibers could not be achieved by electrospinning. This may be attributed to the agglomeration of the excessive ZnO

nanoparticles, which destroyed the continuity of electrospinning. Figure S1d displayed the SEM image of the short precursor fibers. These short fibers were not further calcinated.

According to the series of experiments results, we find that the optimized ZnO dosage was 0.1 g in 1 mL TEOS.
